# Supplementary material for: Farm Animal Welfare during Transport and at the Slaughterhouse: Perceptions of Slaughterhouse Employees, Livestock Drivers, and Veterinarians
Source: Animals (Basel). 2024 Jan 29;14(3):443. doi: 10.3390/ani14030443 (PMC10854723; doi:10.3390/ani14030443)
Supplement: Supplementary file 1 [file animals-14-00443-s001.zip › animals-2781471-supplementary.pdf]

AWQ

Consecution number
/

	GENERAL INFORMATION (personal data will stay anonymous)	DATA	
1	Data of the slaughterhouse visit and time spent.		min.
2	Name and surname of the expert questioner (1) and assistant (2)	**1**	**2**
3	Name and surname of the respondent ***food busines operator or livestock driver***		
4	Approval number		
5	Adress		
6	Animal species alowed to slaughter		
7	Slaughter plan fort the day of visit		
8	Size of the usable floor area of in the slaughterhouse lariage (m^2) and height (m)		
9	Other animals in the slaughterhouse arrea (cat, mice, rat, crow, other birds, etc.)		
10	Species and number of animals present in the slaughterhouse at the time of the visit		

	GENERAL INFORMATION (personal data will stay anonymous)			DATA	
1	**Gender**	**Year of birth**		Male	Female
2	Highest level of education achived?	•	Primary school		
		•	High school		
		•	Grammer school		
		•	Degree (professional or post-secondary programs, 1st level)		
		•	Master's degree (2nd level)		
		•	Unified master's program		
		•	Scientific master's degree		
		•	PhD		
3	What is your current employment?	•	Farmer		
		•	Lodger, fisherman, hunter		
		•	Craftsman, production worker		
		•	Salesman, other service profession		
		•	Entrepreneuer		
		•	Office worker		
		•	Employee in education, health care, engineer		
		•	Senior official, manager		
		•	Unemployed		
		•	Retired		
4	***If other, please specify.***	•			
	What is your field of education?	•	Agricultural		
		•	Veterinary		
	If other, please specify.	•			

GENERAL OPINION OF THE RESPONDENT ABOUT ANIMAL WELFARE (SLAUGHTRHOUSE/DRIVER)

Range: 1 absolutely don't agree **2** dont't agree **3** undecided **4** agree **5** totaly agree

		POINTS				
1.	It is important to have the latest information on animal welfare.	1	2	3	4	5
2.	Public opinion should have no influence on ensuring the welfare of farm animals.	1	2	3	4	5
3.	Farmed animals should live in an environment similar to their natural environment.	1	2	3	4	5
4.	Farm animals are important because of income.	1	2	3	4	5
5.	Farm animals can't show feelings towards humans.	1	2	3	4	5
6.	I think it is important to know the legislation on animal welfare.	1	2	3	4	5
7.	In Slovenia, public opinion on animal welfare is stronger than in other EU countries.	1	2	3	4	5
8	Farm animals should be able to live out their natural behavior.	1	2	3	4	5
9.	Good agricultural management should be a priority for every farmer.	1	2	3	4	5
10.	A farm animal learns more when it is punished than when it is rewarded.	1	2	3	4	5
11.	Everyone involved in meat production should be familiar with animal welfare, including the farmer's family.	1	2	3	4	5
12.	Organizations that advocate animal welfare don't help farmers.	1	2	3	4	5
13.	Stables, including slaughterhouses pens, should be cleaned daily.	1	2	3	4	5
14.	The importance of livestock should be calculated in terms of economic value and cost.	1	2	3	4	5
15.	What people consider as cruelty to animals is only a game between humans and animals.	1	2	3	4	5
16.	There should be practical training on how to ensure animal welfare in practice.	1	2	3	4	5
17.	The government should provide financial support to improve animal welfare in slaughterhouses.	1	2	3	4	5
18.	Farm animals should be housed in accommodation suitable for them.	1	2	3	4	5
19.	Farm animals are like machines (in the sense of effect and efficiency).	1	2	3	4	5
20.	Farm animals obey only when they fear the owner or the slaughterhouse staff.	1	2	3	4	5
21.	I would like to read a booklet on farm animal welfare.	1	2	3	4	5
22.	In today's world, we are faced with so many problems that public opinion about animal welfare is overrated.	1	2	3	4	5
23.	Farm animals have a lower pain threshold than other animals (e.g. dogs, cats, etc.).	1	2	3	4	5
24.	For the farmer it would be better to execute sick animals himself than to pay high costs for treatment or euthanasia.	1	2	3	4	5
25.	The bigger is the farm animal rugher you must be.	1	2	3	4	5
26.	Animal welfare should be taught in elementary and high school.	1	2	3	4	5
27.	The larger an animal is, the meaner it is	1	2	3	4	5
28.	Legal definition (March 2020), animals are sentient beings. Is correct.	1	2	3	4	5
29.	The owner of the farm animal can treat it as he wants.	1	2	3	4	5

GENERAL OPINION OF THE RESPONDENT ABOUT ANIMAL WELFARE (FBO/DRIVER)

Range: 1 absolutly don't agree **2** don't agree **3** undecided **4** agree **5** totaly agree

	DESCRIPTION – general welfare	POINTS				
A	KNOWLEDGE WHAT IS ANIMAL WELFARE					
1.	Laws and regulations concerning animal welfare are good and apply to all animal species.	1	2	3	4	5
2.	If the animals grow, it means that their welfare is taken care of.	1	2	3	4	5
3.	When animals are breading normally, we meet animal welfare standards.	1	2	3	4	5
4.	When animals are in good physical condition, we can say that their welfare is taken care of.	1	2	3	4	5
5.	Due to domestication, farm animals have different needs than their naturally living ancestors.	1	2	3	4	5
6.	The Slovenian Rural Development Project, which includes welfare measures to protect cattle, small ruminants and pigs, is inappropriate and insufficient.	1	2	3	4	5
B	WATER AND FEED REQUIREMENTS					
1.	Feed is not important for the welfare of animals, but only for their growth and development.	1	2	3	4	5
2.	Animals in slaughterhouse lairage do not need drinking water or additional feed.	1	2	3	4	5
3.	Farm animals should have drinking water available at all times.	1	2	3	4	5
4.	Farm animals do not need beading in the slaughterhouses lairage.	1	2	3	4	5
5.	It is important how we keep the feed and that we change beading daily.	1	2	3	4	5
6.	Water contaminated with animal feces does not harm animals.	1	2	3	4	5
C	ANIMAL WELLBEING					
1.	Farmed animals are sentient living beings.	1	2	3	4	5
2.	Animals in slaughterhouse stables don’t feel the need for contact with their fellow species in the stables of the slaughterhouses.	1	2	3	4	5
3.	Animal welfare is important from the birth of an animal until its death. It is important that we care for the welfare of the animal during this time.	1	2	3	4	5
4.	Ammonium irritates the mucous membranes only in humans, it does not bother animals.	1	2	3	4	5
5.	Mobile slaughterhouses mean less suffering for the animals because there is no need for transport, which is very stressful for the animals.	1	2	3	4	5
6.	Slaughtering animals in slaughterhouses has more advantages than slaughtering them on the farm.	1	2	3	4	5
D	HEALTH STATUS					
1.	The presence of disease does not affect the wellfare of animals.	1	2	3	4	5
2.	It does not matter if we vaccinated the animal before slaughter.	1	2	3	4	5
3.	Animals that are healthy can not suffer or experience stress.	1	2	3	4	5
4.	The factors that lead to stress are the same in humans and animals.	1	2	3	4	5
5.	The animals cannot have a stomach ulcer.	1	2	3	4	5
6.	The term biosecurity means that the feed for the animals is hygienically irreproachable.	1	2	3	4	5
7.	The presence of disease does not affect meat production.	1	2	3	4	5

AWQ/SL

SLAUGHTERHOUSE–SPECIAL PART related to the viewing of the lairage (filed by experts in lairage)

**1** – major deficiencies (immediate action required)		**2** – deficiencies warranting (a warning)
**3 –** minor deficiencies (advice required)	**4 –** no deficiencies (compliant with standard)	**5 –** no deficiencies (above standard)

	GENERAL IMPRESSION	POINTS				
1	Upon entering the lairage, the employee announces his arrival/speaks in a friendly manner. **Observe the employee at work.**	1	2	3	4	5
	**ANIMAL BEHAVIOR**					
1	The animals are curious and show exploratory behavior, no aggression is observed between the animals.	1	2	3	4	5
2	*The animals are visibly frightened of the employees/strangers. **Underline the correct answer.***	1	2	3	4	5
3	Handling animals is inappropriate (rough, unprofessional – point 1). **Observe the employee at work.**	1	2	3	4	5
	**LAIRAGE CONDITIONS**					
1	The stocking density in the pens is too high (observation).	1	2	3	4	5
	**ENVIRONMENTAL CONDITIONS**					
1	There are no thermometers and hygrometers, or too few of them. **Underline the correct answer.**	1	2	3	4	5
2	There is a lot of dust in the lairage.	1	2	3	4	5
3	There is a lot of humidity in the lairage.	1	2	3	4	5
4	The smell of ammonia is strongly perceptible in the lairage.	1	2	3	4	5
5	The ventilation and heating are not arranged according to the regulations.	1	2	3	4	5

SLAUGHTERHOUSE – SPECIAL PART related to the attitude towards aminals (employee answers the questionnaire after viewing the lairage)

How important do you think it is … ? **1** not important at all, **2** not important, **3** undecided, **4** it is important, **5** it is very important.

	GENERAL STATUS	POINTS				
1	… to address the animals when you enter the lairage?	1	2	3	4	5
	**ANIMAL BEHAVIOR**					
1	… whether animals are less curious or more aggressive?	1	2	3	4	5
2	… whether animals are afraid in your presence, in the presence of a stranger?	1	2	3	4	5
3	… to move the animals as easily as possible and with as little stress as possible?	1	2	3	4	5
	**LAIRAGE CONDITIONS**					
1	… the number of animals per unit area is?	1	2	3	4	5
	**ENVIRONMENTAL CONDITIONS**					
1	… to know the temperature and humidity in the lairage? ***Circle what the breeder thinks is more important: temperature or humidtity.***	1	2	3	4	5
2	… that there is not too much dust in the lairage?	1	2	3	4	5
3	… that there is not too much humidity in the lairage?	1	2	3	4	5
4	… that there are no unpleasant odors in the lairage?	1	2	3	4	5
5	… that the ventilation and heating in the lairage are regulated/arranged?	1	2	3	4	5

AWQ/D

TRANSPORT –SPECIAL PART related to the viewing of the transport vehicle (compleated by the experts during observation)

1 major deficiencies (immediate action required)
2 deficiencies warranting (a warning)
3 minor deficiencies (advice required)
4 no deficiencies (compliant with standards)
5 no deficiencies (above-standard)

		POINTS				
	**GENERAL IMPRESSION**					
1	When the vehicle arrives, the driver announces his coming/speaks in a friendly manner. ***Observe the work.***	1	2	3	4	5
2	Animals are ditry.	1	2	3	4	5
	**ANIMAL BEHAVIOR**					
1	The animals are curious and show exploratory behavior, no aggression is observed between the animals.	1	2	3	4	5
2	The animals are visibly afraid of the driver/strangers.	1	2	3	4	5
	**TRANSPORT CONDTIONS**					
1	The stocking density on the vehicle is too high (an observation).	1	2	3	4	5
	**ENVIRONMENTAL CONDITIONS**					
1	There are no thermometers and hygrometers on the vehicle, or too few of them. ***Underline the correct answer.***	1	2	3	4	5
2	The ventilation of the transport vehicle is not arranged according to the regulations.	1	2	3	4	5
	**REGULATION COMPLIANCE** (ES 1/2005)					
1	On the driver's statement the date and time of departure is indicated.	1	2	3	4	5
2	The animals were transported to the slaughterhouse without unnecessary stops.	1	2	3	4	5
3	The animals are unfit for transport.	1	2	3	4	5
4	The driver refuses to transport the animals if they are not fit for transport.	1	2	3	4	5
5	The driver of the livestock has a license according to the Regulation (ES) 1/2005	1	2	3	4	5
6	The driver has not received any penalty for the violation of the Regulation (ES) 1/2005.	1	2	3	4	5

TRANSPORT –SPECIAL PART related to the attitude towards aminals (driver answers the questionnaire after viewing the vechicle)

How important do you think it is … ? **1** not important at all, **2** not important, **3** undecided, **4** it is important, **5** it is very important

		POINTS				
	**GENERAL STATUS**					
	… to approach the animals calmly when you approach the vehicle?	1	2	3	4	5
	… that animals are not dirty?	1	2	3	4	5
	**ANIMAL BEHAVIOR**					
	… for animals to be less curious or more aggressive??	1	2	3	4	5
	… whether animals are afraid in your presence, in the presence of a stranger?	1	2	3	4	5
	**TRANSPORT CONDITIONS**					
	… the number of animals in the vehicle is?	1	2	3	4	5

	**ENVIRONMENTAL CONDITIONS**					
	… to know the temperature and humidity in the vehicle?<br>***Circle which the driver thinks is more important: Temperature or humidity.***	1	2	3	4	5
	… that the vehicle's ventilation is regulated?	1	2	3	4	5
	**REGULATION COMPLIANCE** (ES 1/2005)					
1	… to put the correct date and time of departure on your driver's statement?	1	2	3	4	5
2	… to transport animals to the slaughterhouse without unnecessary stops?	1	2	3	4	5
3	… that the animals are fit for transport?	1	2	3	4	5
4	… that you can refuse to transport the animal if it is not fit to be transported?	1	2	3	4	5
5	… that you have a licence under Regulation (ES) 1/2005?	1	2	3	4	5
6	… that you have not been fined for a violation of Regulation (ES) 1/2005?	1	2	3	4	5
